# Knowledge, use and ecology of golden thistle (*Scolymus hispanicus *L.) in Central Spain

**DOI:** 10.1186/1746-4269-5-42

**Published:** 2009-12-22

**Authors:** Sandra Polo, Javier Tardío, Ainhoa Vélez-del-Burgo, María Molina, Manuel Pardo-de-Santayana

**Affiliations:** 1 Departamento de Biología (Botánica). Universidad Autónoma de Madrid. c/Darwin 2. Campus de Cantoblanco. E-28049 Madrid, Spain; 2 Instituto Madrileño de Investigación y Desarrollo Rural, Agrario y Alimentario, Apdo. 127, 28800 Alcalá de Henares, Madrid, Spain; 3 Laboratori de Botànica, Facultat de Farmàcia, Universitat de Barcelona, Av. Joan XXIII, s/n. 08028 Barcelona, Spain

## Abstract

**Background:**

This paper assesses the current ethnobotanical knowledge, use and management of *Scolymus hispanicus *L. in two localities of Central Spain and the relation with its natural abundance. It also addresses the influence of sociodemographic factors such as age, gender and time living in the village in the variation of knowledge and practice levels.

**Methods:**

During 2007 and 2008, 99 semi-structured interviews and a questionnaire were made to a random stratified sample by sex and age, asking them about their traditional knowledge and practices (use and gathering) of *Scolymus hispanicus*. A knowledge and practice (KP) index was created based on the answers to the questionnaire.

**Results and Discussion:**

*Scolymus hispanicus *is still gathered and consumed by 20% and 35% of the informants, respectively. According to the KP index, the knowledge and practice level is similar in both villages. Age and time living in the village are the factors that better explain the variability in the KP level. People living for more than ten years in the village and those older than 60 years have the highest knowledge level, whereas the younger than 19 the lowest.

**Conclusions:**

Our data suggests that the prevalence of ethnobotanical knowledge and uses depends more on the cultural importance of the plant and the transmission of such popular knowledge than on the resource's abundance.

## Background

Along the last 30 years Spain has undergone an outstanding development in ethno-botanical research [1-5]. These studies have principally focused on compiling the ethnobotanical knowledge, use and management of elderly people, which hold most of these knowledge and practices. Nevertheless, and despite the great interest shown by quantitative studies that evaluate the individual ethnobotanical knowledge [6], only a few of such studies have been carried out in Spain [7,8]. To assess the current individual level of knowledge and practices, it is necessary to interview not only the elderly group but also other social groups such as children, young people or immigrants.

Several indexes based on objective tests have been used for measuring the traditional ecological knowledge. Some encompass both knowledge and practices because previous research suggests that both dimensions do not necessarily overlap [6]. They help analyzing the changes in the level of the ethnobotanical knowledge and practices held across generations, and may explain which variables are responsible for its preservation or abandonment [6,9].

Likewise, information obtained from ethnobotanical interviews can be complemented with ecological studies. The combined analysis of ecological and cultural factors which determine the use of the species allows to study the relationship between the availability of the resources and its cultural importance. It is also helpful for studying the influence of the exploitation of the resource in its distribution and abundance [10-12].

Wild edible plants have been important food resources during scarcity periods and as dietary supplements, providing trace elements, vitamins and minerals. Nowadays, they are consumed in many countries for the pleasure of gathering them, recreating traditional practices and enjoying characteristic flavours, more than for their nutritional value [12-18]. That is the case of the golden thistle, *Scolymus hispanicus *L. (Compositae), a prickly perennial herb with a circum-Mediterranean distribution which grows all over Spain but it is scarce in the north of the country. It can be found in uncultivated agricultural fields, weed areas, roadsides, waste places and nitrified lands [19,20]. Another species of the same genus, *S. maculatus *L., coexists with *S. hispanicus *in some warm areas of southern Spain. Although the former is an annual herb, both look quite similar at the gathering time and it is likely that both are used in the same way in some provinces of Andalusia. However there is only one well-documented citation of the edible use of *S. maculatus *from Jaén [21].

**Figure 1:**
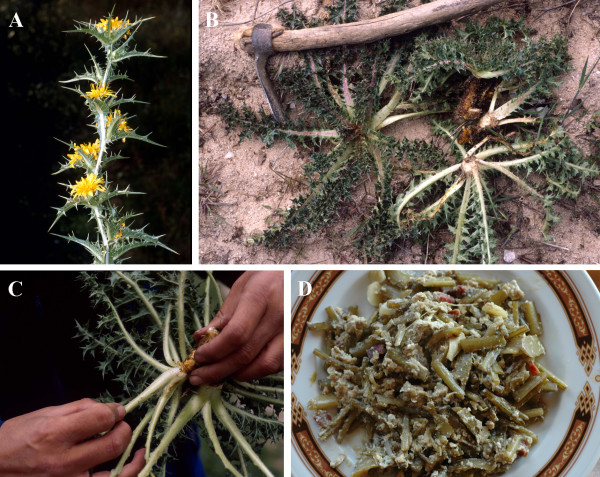
***Scolymus hispanicus *specimens: blooming plant (a), small rosettes collected (b), peeling labour (c), Spanish traditional dish with *cardillo***.

The Golden Thistle, locally known as *cardillo*, is one of the most appreciated wild vegetables in Central Spain [2] and other Spanish regions [22]. It is also consumed in other Mediterranean countries, such as Portugal [23], Morocco [24], France [25], Italy [26], Greece [27], Cyprus [28] and Turkey [29]. It can be said that *cardillo *plant is consumed in almost all the places where it grows. The part used for consumption is the central nerve of their prickly basal leaves. They are boiled and then usually lightly fried with a bit of garlic, cured ham and scrambled eggs (Figure 1). In addition, its root has been employed as a coffee substitute and its flowers as a colouring alternative to saffron [2,30]. It has many medicinal properties such as diuretic, depurative, digestive, choleretic and lithiuretic [3,31,32]. The great gastronomical interest of this species has led to the study of its agronomical potential [33].

Given the cultural importance of this species, a comparative ethnobotanical survey was conducted in order to analyze its current use and knowledge, as well as to describe the relation with its natural abundance in two different areas of the province of Madrid (Central Spain). Specific aims of this research were:

1. To describe the ethnobotanical knowledge about *cardillo *plant in the selected villages of Brea de Tajo and Canencia.

2. To compare the distribution and maintenance of ethnobotanical knowledge and practices in both localities.

3. To estimate the density and abundance of *cardillo *plant in the traditional gathering sites of both areas.

Moreover, we wanted to test the following hypotheses:

a. The knowledge and practice level of *cardillo *plant is higher in elderly people.

b. Knowledge and consumption level is supposed to be higher in the areas where the plant is more abundant.

## Methods

### Study sites

**Figure 2:**
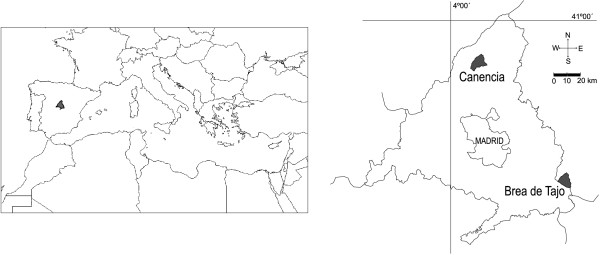
**Localization of the surveyed areas in the province of Madrid**.

Brea de Tajo and Canencia were the two chosen localities from the province of Madrid (see Figure 2). They were selected because the current gathering and consumption of the species had been recently described [20]. Their differences in altitude, rainfall, lithology and land uses offered an opportunity to analyze whether these factors might have influenced the abundance and use of the species.

Brea de Tajo municipality is located in the Southeast of Madrid province, 65 km far from the city of Madrid (Figure 2). The average altitude is 715 m and it has an extension of 44.3 km^2 ^[34]. Its rich soils are developed on basic rocks (limestone, marl, or gypsum). It has a dry continental Mediterranean climate, with average annual temperatures of 13-14°C and average annual rainfall of 400-500 mm [35]. Land is mainly devoted to dry agriculture, being cereals, grapevine, almond and olive trees the most important crops.

Canencia is located in the North mountainous area of the province, 70 km far from the city of Madrid (Figure 2). It has an average altitude of 1150 m and an extension of 53.5 km^2 ^[36]. Its oligotrophic soils are developed on metamorphic rocks. It has a sub-humid continental Mediterranean climate with average annual temperature of 9-10°C and average annual rainfall of 800-900 mm [35]. The land is chiefly dedicated to livestock farming of sheep and cows that feed in pastures, *piornales *(mountain shrub areas) and *cervunales *(higher dense lawns) [37].

**Figure 3:**
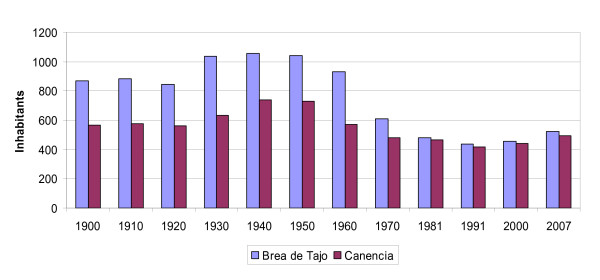
**Historical demography in Brea de Tajo and Canencia (INE 2009, http://www.ine.es.)**.

The demographic evolution during the last decades has been very similar in both localities (Figure 3). The same maximum population rate can be appreciated in the mid of the last century and a minimum rate of population at the end of the century. There were important socioeconomic changes during the second half of last century due to the industrial development of the country. These changes led to a massive migration from rural areas to cities, causing a progressive drop of the population up to the beginning of the XXI century. Population has gone up along recent years thanks to migrant people from urban areas and other countries (15% in Canencia and 6% in Brea de Tajo [38]).

**Figure 4:**
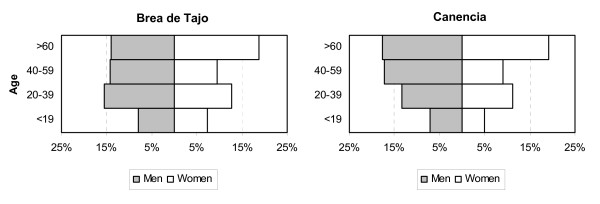
**Current population of Brea de Tajo and Canencia organized by sex and age group (INE 2009, http://www.ine.es.)**.

As can be appreciated both in Figure 4 and in Table 1 figures, both localities have an outstandingly aged population (stressed in the village of Canencia). It is due to the increase of life expectancy, birth rate decrease and the migration from rural areas by an important working population group (young people).

**Table 1:** Population of each village divided by age and sex

	Brea de Tajo			Canencia		
Age group	Men	Women	Total	Men	Women	Total
**<19**	42 (4)	38 (4)	80 (8)	35 (3)	25 (2)	60 (5)
**20-39**	81 (8)	67 (7)	148 (15)	66 (7)	56 (6)	122 (13)
**40-59**	74 (7)	50 (5)	124 (12)	85 (8)	45 (4)	130 (12)
**>60**	73 (7)	98 (9)	171 (16)	88 (9)	95 (9)	183 (18)
**Total**	270 (26)	253 (25)	523 (51)	274 (27)	221 (21)	495 (48)

In brackets the number of informants interviewed

### Methodology

With the aim of having a complete overview of the current knowledge, use and management of *cardillo *plant, as well as the species distribution and abundance in the study sites, ethnobotanical and ecological research techniques were combined. On the one hand, semi-structured interviews and a questionnaire about its use, knowledge and traditional management were conducted [10]. On the other hand, to estimate the current abundance and density of the species in both localities, plant sampling techniques were employed [11,39].

#### Ethnobotanical Knowledge and Practices

During November 2007 to April 2008 independent interviews were carried out divided in two distinct parts. The first part consisted in a semi-structured interview and the second part included a questionnaire. Through semi-structured interviews we obtained detailed information about a) the traditional knowledge and practices (use and gathering) of *Scolymus hispanicus*, b) the origin of this knowledge and c) the ecology and abundance of the species perceived by locals. We also run a questionnaire for evaluating the individual ethnobotanical knowledge and practices levels about *S. hispanicus *of each informant, consisting of the following checklist of questions:

• Informant personal attributes: sex, age, education level, occupation and number of years living in the village.

• Do you know this plant? Two photographs/images of the plant were shown while asking the question.

• Do you know its name?

• Do you know its uses? If he/she mentioned any, he/she was asked: Which part of the plant is used? How is it prepared and consumed?

• Do you use it nowadays? Did you use it in the past? If he/she used it only in the past, he/she was asked: Why don't you use it anymore?

• Do you gather it nowadays? Did you gather it in the past? If he/she gathered it in the past, but don't gather it anymore, he/she was asked: Why don't you gather it anymore?

Once personal data were taken down, two photographs of the plant were shown to the informant, one of the basal rosette at the gathering time before the flowering stem had sprouted and a second at blooming time. If the person didn't recognize the plant, the Spanish plant name was indicated and the rest of the interview was continued.

A stratified random sampling by sex and age was employed, interviewing 10% of the population of each village. As shown in Table 1, the number of individuals of each stratum was balanced to the real proportions of each group in the whole populations. For example, in Brea de Tajo four male informants under 19 years were interviewed, 10% of the 42 male inhabitants of that age group. A total amount of 51 were interviewed in Brea de Tajo (mean age 45.2 ± 3.2; 49% women) and 48 informants in Canencia (mean age 50.2 ± 3.5; 44% women). As shown in Table 1, four age groups were selected, each of them can be considered as a different generation: "grandparents" (≥ 60), "parents" (40-59), "children" (20-39) and "grandchildren" (≤ 19). Only people who were living in the village for more than one year were interviewed, since it is the minimum period for having experienced all the climatic, phenological, agricultural and social events that occur during a complete annual cycle.

A knowledge and practice (KP) index was created based on the questionnaire prepared to evaluate the individual ethnobotanical knowledge and the past and present use and gathering levels of *S. hispanicus*. The value of the index was the sum of the score given to the answers. Each question could sum 0.25, 0.5 or 1 point, according to the rules shown in Table 2.

**Table 2:** Scores given to the answers that form the KP index.

Recognizes the plant in the photographs	0.25
Knows the name	0.25
Knows the use, part of the plant used, mode of consumption, etc	0.50
Uses it at present	1.00
Used it in the past	1.00
Harvests it at present	1.00
Harvested it in the past	1.00

The first three questions deal with theoretical knowledge of the plant that do not entail putting it into practice, and we decided that they should sum 1 point. Other issues related to the present or past practical knowledge of the plant (consumption and gathering) by the informant have a higher weight. This index theoretically varies from 0, when the informant does not know anything about the plant, to 5 in the case that the informant recognizes the plant in the photographs, its name and uses, and have ever collected and consumed it in past and present times.

Once the KP index of every single informant was calculated, differences among the mean values of localities, age groups, sex and time living in the locality were statistically analyzed. Taking into account that the variable is not normally distributed (Kolmogorov-Smirnov test) and that there was not variance homogeneity among groups (Levene test), non-parametrical tests were employed. Kruskal-Wallis test was used to confirm the existence of differences among groups and the means were compared two by two using Mann-Whitney test [40].

#### Plant Sampling Methods

To evaluate the abundance of *cardillo *plant, the area around the places where informants traditionally gather them within a 2 km radius of the villages were visited with the help of the informants. Plots were established in these gathering areas after checking that the plant did not grow in other areas. Density was estimated using 220 transects of 20 × 1 m randomly located in the gathering sites. The number of transects per plot was proportional to the surveyed areas. The sampling took place between the end of September and the beginning of October 2007, when the specimens were easily counted thanks to the size of the dried inflorescences that were still linked to the rosette and root.

In Canencia 7.6 ha were sampled in four plots of land (180 transects). All of them were located in grazing lands that were long ago cultivated with cereals, reaching an average altitude of 1228 m. In Brea de Tajo, only one plot was found in an abandoned agricultural field. Therefore, only 0.85 ha (40 transects) were sampled with an average altitude of 744 m.

Since Kolmogorov-Smirnov test proved that the variable "density" did not follow a normal distribution, the differences of *cardillo's *density between both localities were analysed with the non-parametric MannWhitney U test.

## Results and Discussion

### Ethnobotanical Knowledge and Practices

The ethnobotanical information obtained in the interviews was organized and analysed around two main subjects. Firstly, we describe the knowledge and practices of *Scolymus hispanicus*, such as the local uses, the perceived ecological preferences of the species, the gathering customs and the origin of the knowledge. Secondly, we evaluate the present structure of this traditional knowledge and practices according to age, gender and time living in the village.

Table 3 shows a summary of the results of the questions used for obtaining the knowledge and practice (KP) index divided by age groups in both studied sites.

**Table 3:** Number of positive respondents to each question divided by age groups.

	Brea de Tajo					Canencia					Total
	**≤19**	**20-39**	**40-59**	**≥60**	**Total**	**≤19**	**20-39**	**40-59**	**≥60**	**Total**	
	
Interviewees	8	15	12	16	51	5	13	12	18	48	**99**

Recognized the plant	4	14	6	16	40	2	2	10	18	32	**72**

Knew the name	3	13	11	16	43	2	3	12	18	35	**78**

Knew its uses	3	13	10	16	42	2	3	12	18	35	**77**

Use it at present	0	1	4	12	17	2	1	6	9	18	**35**

Used it in the past	0	7	7	16	30	2	2	11	18	33	**63**

Gather it at present	0	1	3	4	8	0	1	3	7	11	**19**

Gathered it in the past	0	9	3	12	24	0	1	10	12	23	**47**

#### Knowledge about Biological and Ecological Characteristics of the Plant

As can be seen in Table 3, 72 out of 99 informants (73%) recognized the plant in the photographs. Nevertheless, once the species' name was mentioned to them, we concluded that a lightly higher proportion of informants, 79% (78 out of 99) knew it. There were people who knew the plant but could not recognize it in the photographs, as was described by Thomas *et al*. [41]. The percentage of people who recognized the plant or knew the name was slightly higher in Brea de Tajo (78% and 84%) than in Canencia (67% and 73%). It is clearly a generalized knowledge. Only children, immigrants from other countries, people from the city who have just moved to the village and a few native people, less than 45 years old, did not recognize the plant.

Regarding *cardillo's *habitat, just 58 of the interviewed informants (57%) were able to describe some characteristics (Table 4). All of them had consumed it at any time in their lives. Many informants in both localities (81%) stressed that it grows along roadsides and pathsides, in unstable, loose and poor soils. Although 69% of Brea de Tajo informants said that it used to grow in farming fields, both cultivated and fallow lands, just 7% of informants mentioned such factor in Canencia. On the other hand, livestock presence (45%) and water availability (41%) were considered more important for the informants of this locality. They highlighted that spring rains were essential for its growth. However, only three people mentioned that factor in Brea de Tajo. Those big differences are due to the different land uses between both localities. In Brea de Tajo, a mainly agricultural village, *cardillo *habitat has always been linked to cultivated lands. However, in Canencia livestock has always been more important than agriculture, especially during the last three decades. This has also influenced the current *cardillo *distribution.

**Table 4:** Number of informants who mentioned *cardillo *ecological characteristics in each locality.

	Brea de Tajo	Canencia	Total
Number of informants who mentioned habitat characteristics	29	29	58
Roadsides, loose soils, poor soils	24 (83%)	23 (79%)	47 (81%)
Farming fields, both cultivated or fallow lands	20 (69%)	2 ( 7%)	22 (38%)
Water, humidity, rains	3 (10%)	12 (41%)	15 (26%)
Livestock	0 ( 0%)	13 (45%)	13 (22%)

Number of informants who mentioned historical abundance differences	24	24	48
There are now less plants than before	21 (88%)	5 (21%)	26 (54%)
There are no differences between past and present abundance	3 (120%)	19 (79%)	22 (46%)

Mainly due to these reasons, there were clear differences about the abundance perception of *cardillo *plant among the 24 informants of each locality who answered the question. A big majority of the informants of Brea de Tajo (88%) said that there is currently even less *cardillo *plant than before, and just some people (12%) did not find any difference (Table 4). Furthermore, these last three informants were not a reliable source since they had not gathered *cardillo *in the last years. They mentioned two reasons for this decrease: the use of herbicides and the use of tractors for the plough, both modern agricultural practices. "There were more [*cardillos*] long ago because we ploughed with mules, and now since we use tractors we plough deeper. And also because we did not use poison in our soils, and now we do". These results corroborate the hypothesis previously discussed. In this mainly agricultural locality, *cardillo *distribution is highly associated with cultivated lands and has been strongly affected by changes of modern agriculture. Currently it is consigned to roadsides or abandoned agricultural lands.

Nevertheless, 79% of Canencia's informants agreed that there were no differences between the abundance in the past and nowadays. Just 21% of the informants disagreed, arguing that it was not only due to the lack of rain ("there is much less *cardillos *now than before as it rains much less as well, and weather does not come as it should"), but also due to the agricultural fields disappearance ("there were more *cardillos *long ago, because they used to grow inside the crop lands"). In fact, in this livestock village, Golden Thistle also grew in the past as a weed of crop lands, which are now pastures. They were usually gathered when these crops were hand-weeded. However, in spite of these land use changes, *S. hispanicus *have not disappeared.

#### Uses

Informants of both communities only mentioned the edible use of *cardillo*. As could be expected and it is shown in Table 3, the number of people that knew the use of the plant was a bit higher (77; 78%) than the number of informants that only recognized the plant (72; 73%). There are people that, although they do not know how the plant is, they have consumed it or, at least, they have heard that it is an edible plant. The percentage was slightly higher in Brea de Tajo (73%) than in Canencia (67%). The most widespread mode of consumption in both villages (62%) was as a garnish for *cocido*, a traditional Spanish dish with stewed chickpeas, potatoes, and meat. The peeled basal leaves are previously boiled and then fried lightly in olive oil with a bit of garlic. In Brea de Tajo, 37% of the informants also mentioned the same mode of preparation but they consumed the plant as a main course called "*cardillo rehogado con ajos*". In Canencia, the second most mentioned mode of preparation (58%) was made with scrambled eggs or in an omelette. Other modes of preparation cited were: fried with cured ham, tomatoes, in a salad, etc. They are also boiled and preserved in cans or frozen to consume them along the year. Furthermore, an informant from Brea de Tajo said that it had also been locally marketed: "I know them thanks to my uncle since he is fruit seller and sometimes they were sold in his shop".

Regarding its preparation, all the informants agreed that only women did cook them. Several men mentioned that they had stopped gathering and consuming *cardillos *since there was no female presence at home.

As shown in Table 3, a great majority of the interviewees of both villages (nearly 60% and 70% in Brea de Tajo and Canencia respectively) used to eat them in the past. Nevertheless, only a bit more than half of them still do it (33% and 38% of the respondents respectively).

According to the informants, *cardillo *plant was eaten long ago mainly as a nutritional complement in shortage times and it is therefore considered by many as "poor man's food" ("anciently it was food for poor people and now is for rich ones"). Its excellent flavour surely explains why it is currently being gathered, prepared and consumed; "People come even from Madrid to collect them", "anciently we ate them because we needed for surviving; now it is done by whim. That is why weekend trippers harvest them while having a walk".

#### Gathering

*Scolymus hispanicus *specimens are generally gathered from April to May, depending on the rain fall along the year. As an informant from Brea de Tajo told us: "*los de abril para mí, los de mayo para mi amo y los de junio para mi burro" *(April ones are for me, those from May for my boss and those from June for my donkey).

Gathering and peeling are the hardest task of the process. It is an activity mainly related to men "since these plants prick a lot". However, some interviewed people stated that women picked them up too. In fact, 16 out of 47 people who gathered them in the past, and 8 out of 19 people who still gather it, were women (Table 3). Such gender distribution is similar in other regions [e.g. [42-44]].

Although the percentage of people who gathered this plant long ago was similar in both villages (47% and 48% respectively), 23% of people from Canencia still does it and only 16% in Brea de Tajo (Table 3). The main reason why they have stopped picking them up is that nowadays they spend much less time in the field than in the past. Only 19% out of 35% people who eat *cardillo *plants also gather them. The rest of informants merely consume it when eating at their parent's home or when a friend or relative gives them the plant. These goods interchanges are very valued locally and they reinforce social and familiar links.

#### Origin of the knowledge

According to the interviewed people, the knowledge needed to recognize, collect and prepare *cardillo *plant was mainly transmitted from parents or other close relatives like grandparents, brothers or sisters (89% of Brea de Tajo informants and 78% of Canencia). The family is very important in the process of ethnobotanical knowledge acquisition [45] since it shapes the values about the natural environment in which the family lives. Friends were also mentioned as knowledge transmitters (11% in Brea and 22% in Canencia). They are especially important during childhood, since children have enough free time to explore the land and learn while playing [46].

### Distribution of ethnobotanical knowledge and practices

In this section we analyze the differences in individual knowledge and practices about *cardillo *according to the KP index. We specifically analyze if sociodemographic characteristics such as age, gender and time living in the village could explain why these differences appear.

#### Differences of ethnobotanical knowledge and practices between Brea de Tajo and Canencia

As can be observed in Table 5 the mean values of KP index for the informants of both villages are very similar: 2.35 for Brea de Tajo and 2.44 for Canencia. The nonparametric Mann-Whitney U Test (p = 0.997) shows that this difference is not significant.

**Table 5:** KP index value of the different age groups in the two surveyed localities (mean and standard error)

	≤19	20-39	40-59	>60	Total
Brea de Tajo	0.41 ± 0.16 aA	2.02 ± 0.36 bA	2.19 ± 0.54 bA	3.75 ± 0.21 cA	2.35 ± 0.24 A
Canencia	0.80 ± 0.58 aA	0.60 ± 0.40 aB	3.46 ± 0.38 bA	3.56 ± 0.29 cA	2.44 ± 0.27 A
Total	0.56 ± 0.23 a	1.36 ± 0.29 a	2.82 ± 0.35 b	3.64 ± 0.18 b	2.39 ± 0.17

Means followed by different letters differ statistically (Man-Whitney U test, P < 0.05). Small letters compare differences in the same locality (rows) and capital letters in the same age group (columns).

According to the adopted punctuation system, the average inhabitant has a medium KP index level in both localities. It means that they were not only passive knowledge holders, since they recognized the plant and knew its uses, and also have ever consumed or gathered *cardillos *at any time in their lives.

#### Differences among age groups

Distribution of mean values of KP index in the different age groups in both localities can be seen in Table 5. As shown in this table, there are significant differences among age groups but they are not the same in each village.

In Brea de Tajo, Kruskal-Wallis test showed significant differences among the KP index averages of the age groups (χ^2 ^= 23.30; p = 0.00). Mann-Whitney tests showed that differences among the age groups 20-39 and 40-59 years are not significant (p = 0.87), but are slightly different from that of age groups ≤ 19 (p = 0.002; p = 0.012, respectively) and ≥ 60 (p = 0.001; p = 0.019, respectively). Likewise, KP index difference between ≤ 19 and ≥ 60 groups was also relevant (p = 0.00).

As reflected in Table 5, the group with a higher KP level in Brea de Tajo is the one older than 60 (mean = 3.75). All the informants recognized the plant and knew its uses and ways of preparation, since they had used it at anytime in their lives. It is the gathering group *par excellence *(see Table 3), although most of them do not collect it more due to their age-related problems. It is the group with a better environmental knowledge. All the informants at least at any time in their lives worked as farmers, acquiring an important and deep ethnobotanical knowledge. They suffered the scarcity and hunger period after the Spanish Civil War. They were also witnesses of the huge socioeconomic changes that took place in Spain in the mid-fifties, and even deeper during the seventies. Brea de Tajo changed from traditional agriculture to a mechanized modern agrarian exploitation system. All that brought along an important rural exodus: a big part of the active population moved from the villages to the cities [47].

The knowledge averages of the age groups 20-39 and 40-59 are intermediate (KP index = 2.02 and 2.19, respectively) and differences are not statistically significant. The majority of them knew the plant ant its uses, since they have consumed them mainly in the past although just few of them gather and use it now. Almost all of them were born in the village or have been living there for more than 10 years.

As can be appreciated by the standard error of Table 5, in the group of 40-59 years there is a big variability in the KP index of Brea de Tajo informants. Some are still gatherers and others have just heard about it. This group have distanced themselves from the land as reflected by the statement of one of our interviewees *"I do not like land stuff"*. What they know about the land is just what they heard from parents and relatives and not from their own experience. Almost all the informants were dedicated to the service sector and only some of them had ever worked the land with their family. This generation can be understood as a mixture of rural knowledge persistence and rural exodus. Once the modern agrarian system was established, land working force decreased and participating in land works by every single member of the family was not so necessary [7]. Most of them preferred to find a job in the service sector, which provided them higher salaries. It is also amazing that the 20-39 age group had a knowledge level similar to that of the previous group. The majority had consumed *cardillo *previously and many of them had gathered it and were interested in rural traditional knowledge.

The lowest mean of KP index (0.41) is that of the age group ≤ 19. Half of the informants of this group did not even know the plant and no one had ever consumed or harvested them. Those informants who were born in the village showed a slight better knowledge since three out of five knew the plant while only one of the three people who were not born there knew it.

In Canencia, the other surveyed village, there were also significant differences among KP index averages of the different age groups according to Kruskal-Wallis test (χ^2 ^= 24.84; p = 0.00). Nevertheless, as can be seen in Table 5, differences among age groups show slightly different results from those of Brea de Tajo. Mann-Whitney tests showed that differences between means of age groups ≤ 19 and 20-39 are not statistically significant (p = 0.63), neither between age groups 40-59 and ≥ 60 (p = 0.95). Nevertheless there are relevant differences between the two youngest groups against the two elderly ones (p < 0.001 in all the cases).

As shown in Table 5, the highest KP index figures are in 40-59 and ≥ 60 age groups, without significant differences between its averages (3.46 and 3.56, respectively). The majority of the informants knew the plant and its uses, and had gathered and used it in the past. Many of them still do it nowadays. Almost all of them were born in Canencia and are deeply-rooted in the local traditions.

The *cardillo *knowledge and practice level of the age group ≥ 60 is very similar to that of the same group in Brea. Everyone had worked as a farmer or rancher and they knew its environment very well. A lot of them worked for many years in the building industry or other services. According to Ruiz Sanz [47] the fifties and seventies of the last century brought major developments in stockbreeding. The rapid Spanish economical rise in these decades led to a change in the Spanish population food habits. Meat consumption increased rapidly and was doubled between 1960 and 1974. During this rising demand, industrial farming was entrenched and an important part of the extensive farms in Canencia were substituted by intensive ones. Sheep and goat cattle decreased tremendously since they were not profitable any more, having almost disappeared. Milk and meat production in cattle have significantly increased once entering in an intensive system based on fodder and forage. Most of the autochthonous cattle breeds were substituted by others more productive. Likewise, in the sixties or seventies of the last century those cultivated lands dedicated to staple crops (wheat, rye, potatoes and legumes) almost disappeared [48].

Regarding the 40-59 age group, although most of the informants worked in the service sector, many of them were prominent experts in local rural customs. This indicates that this knowledge still persists and is transmitted and valued.

The KP index level of ≤ 19 and 20-39 age groups is lower than previous groups and the difference between its means (0.80 and 0.60, respectively) is not significant. They basically knew the name and use of the plant "by hearsay".

The low KP index level in the 20-39 age group seems to be linked with the arrival of new active population during the last decade. Canencia has always given a warm welcome to those people from the city that were looking for a more natural environment and also to migrants from other countries that look for better economic opportunities. Four out of 13 people of this group were Spanish people coming from Madrid and Segovia and five of them were migrants of different nationalities (mainly Bulgarian). This low level of KP is probably due to the relevant influx of people who come from cultures that have a slight local environmental knowledge of this region [e.g. [49]].

Finally, the ≤ 19 age group had a bigger variability in the knowledge level. Two out of the three interviewed people born in Canencia had a KP index level quite high; they knew the plant and its uses and they also used it or kept using it due to the influence of the family. Nevertheless, one of those people born in the village and two migrant people did not know the plant.

As in other studies [e.g. [50]], our data suggests that there is an alarming erosion of traditional ethnobotanical knowledge related to *S. hispanicus*. Such erosion can be appreciated in the big knowledge differences among children and young people if compared with elderly people. However, it is also true that a part of these differences are due to the fact that the ethno-botanical knowledge is acquired in the process of growing up [51] and it seems that this age group has not had enough interest or time to assimilate it. The fact that just half of those who gathered and consumed *cardillo *in the past still do it nowadays also shows this alarming erosion.

#### Differences between men and women

The mean KP index values of the informants of both villages separated by sex are shown in Table 6. According to the Mann-Whitney non-parametric test, differences between the means of KP index of men and women were not significant at 0.05 level in both villages (p = 0.16 in Brea and p = 0.95 in Canencia). Significant differences between knowledge level of men (p = 0.61) and women (p = 0.66) in each locality were not found either.

**Table 6:** KP index of men and women in the two surveyed localities (mean and standard error)

	Men	Women
Brea de Tajo	2.66 ± 0.31 a	2.02 ± 0.35 a
Canencia	2.43 ± 0.36 a	2.46 ± 0.43 a
Total	2.54 ± 0.24	2.22 ± 0.27

The same letter mean non-significant differences (P < 0.05)

Although informants mentioned that gathering and peeling was mainly a men activity, the percentages of men and women who gather *cardillos *nowadays are not very different (17% of women and 21% of men). However, the differences were more important in the people who harvested it in the past (35% of women and 59% of men). Despite some gendered differences appear if only practical aspects are considered, KP index of both genders is alike since their theoretical knowledge is very similar.

#### Differences regarding the time living in the village

According with the nonparametric Mann-Whitney test, the knowledge and practice level about *cardillo *plant (KP index) of the inhabitants living less than 10 years in the village is significantly lower than those that had been living there for more than 10 years (see Table 7). The same happens in Brea de Tajo and Canencia (p < 0.001 both).

**Table 7:** KP index of the informants who have been living for less or more than ten years in the two surveyed villages (mean and standard error).

	<10 years	> 10 years
Brea de Tajo	0.21 ± 0.12 a	2.63 ± 0.24 b
Canencia	0.70 ± 0.44 a	3.16 ± 0.26 b
Total	0.55 ± 0.31	2.86 ± 0.18

Different letter means significant difference (P < 0.05)

Eight out of 19 of the informants living less than 10 years in Brea de Tajo or Canencia were migrants from other countries, two of them children. The rest were people mainly coming from Madrid, three of them underage people. All the adult informants worked in the service sector and did not labour in direct contact with nature. Only three of the adults knew the plant and just one gathered and consumed it.

### Density of thistles

Mean values of density and total number of *cardillos *estimated in each village are shown in Table 8. According to Mann-Whitney test, *cardillo*'s density is significantly lower in Canencia (p = 0.01). This difference can be due to the soil type (eutrophic soils in Brea de Tajo and oligotrophic in Canencia) and the land use (abandoned agricultural lands versus lands frequented by cattle, respectively).

**Table 8:** Density and Estimated Total Number of Plants in Each Village.

	Area (ha)	Density (plants/ha)	Estimated total number of plants (area*density)
Brea de Tajo	0.85	750 ± 187 a	637 ± 159
Canencia	7.60	208 ± 29 b	1583 ± 220
Total	8.45	307 ± 43	1411 ± 184

The total abundance of *cardillo *was estimated multiplying the area of each plot by the density of plants of this area. According to these results, although the *cardillo's *mean density is higher in Brea than in Canencia, the estimated total abundance of the gathering sites is higher in Canencia (1583 ± 220) than in Brea (637 ± 159). This is very important for gatherers since the harvesting effort depends not only on the density but also on the total amount of plants available in that area.

These results complement and support the information coming from the interviews regarding the availability perceived by locals. As previously discussed, although in Canencia informants did not appreciate a decrease in the abundance of the plant, in Brea de Tajo almost everyone confirmed that there are currently much less *cardillos *than before. Such availability decrease may be related to the habitat loss due to the introduction of modern agricultural practices.

It should be expected that this lower abundance of *cardillo *plants in Brea de Tajo came along with a lower KP index level in comparison with Canencia's informants. Nevertheless, as stated before, there are no significant differences in the KP index level of both localities. In fact, in Brea de Tajo, where *cardillo *plant is less abundant, people use to go to other areas far from the village to get them. Finally, on the contrary as we hypothesized, it seems that the level of knowledge, use and gathering of *cardillo *do not depend on the local availability of the resource but on cultural aspects as the plant appreciation, which contribute to the maintenance of knowledge and traditional uses [12].

Unfortunately we cannot measure the abundance in the past, since it would be helpful for testing the statement of some informants in Brea who said that the plant is less abundant now than before. The higher abundance of the past could have influenced its current high cultural importance, and therefore promote that people nowadays go to other areas far from the village to harvest it. In this case the hypothesis that the level of KP depends mainly on the local availability of the resource could be true.

## Conclusions

The use of ethnobotanical and ecological sampling methods has been supplementary for the analysis of the individual ethnobotanical knowledge and practices. Highlighted conclusions are:

• Roadsides, loose and poor soils are the main habitat of *cardillo *on both localities. In farming lands, fallow lands or abandoned agricultural lands it grows more densely. Nevertheless, in pastured lands that were previously agricultural lands it does not grow so densely but it is easier available because the plant occupies a bigger area.

• Gathering was carried out traditionally mainly by men while women were in charge of preparing it for its consumption. However, significant differences are not observed in knowledge and practices between men and women.

• The main mode of transmitting this traditional knowledge is oral, thanks to close relatives, chiefly vertical transmission by parents. However horizontal transmission by friends was also mentioned as a way among the elder generations.

• The knowledge and practice index (KP index) is an interesting tool for exploring the distribution of individual ethnobotanical knowledge and practice and for evaluating the prevalence of this edible use.

• The main factors that have an influence in the variability of *cardillos' *KP index are the age group and lifetime living in the village. Though some differences appear between the two villages, as expected, the group ≥ 60 years is the one with the highest knowledge level, whereas the group ≤ 19 has the lowest one in both localities.

• The use of *cardillo *has suffered an important decrease in both localities. It is currently gathered by less than the half of people that did it in past. Nevertheless, it is still gathered and consumed by 20% and 35% of the informants, respectively.

• Although the availability of the plant differs between Brea de Tajo and Canencia, their KP indexes are not significantly different. This result suggests that the prevalence of ethnobotanical knowledge and uses depends more on the cultural importance of the plant and the transmission of such popular knowledge than on the resource's abundance.

## Competing interests

The authors declare that they have no competing interests.

## Authors' contributions

SP and MP designed the field work and SP carried it out. The paper was written by all the authors. All authors read and approved the final manuscript.
